# Remarks on the Recently Proposed American Plan of Treating Gun-Shot Wounds of the Chest by "Hermetically Sealing,"

**Published:** 1864-04

**Authors:** T. Longmore

**Affiliations:** Professor of Military Surgery, Army Medical School.


*Remarks on the Recently Proposed American Plan of Treating Gun-Shot Wounds of the Chest by "Hermetically Sealing."* By Deputy Inspector-General T. Longmore, Professor of Military Surgery, Army Medical School.

A plan of treating chest wounds has been lately brought to notice in the "American Medical Times," by Dr. B. Howard, of the United States Army, which is called by its author the "treatment by hermetically sealing;" and the editor states it to be understood that at the next engagement of the Army of the Potomac an hospital is to be organized, under charge of Dr. Howard, for the sole purpose of treating gun-shot wounds of the chest by the sealing process. Dr. Howard advocates the propriety of this treatment for all penetrating wounds of the chest by gun-shot. He also describes it to be applicable to penetrating wounds of the abdomen, whether made by gun-shot or stabbing instruments.

The following is a description, in Dr. Howard's own words, of the manner in which the operation of hermetically sealing is to be practised:

"All accessible foreign bodies having been removed, introduce the point of a sharp-pointed bistoury perpendicularly to the surface tust beyond the contused portion, and, with a sawing motion, pare the entire circumference of the wound, converting it into a simple incised wound of an elliptical form. Dissect away all the injured parts down to the ribs, then bring the edges of the wound together with silver sutures, deeply inserted, at not more than a quarter of of an inch apart; secure them by twisting the ends, which are then cut off short and turned down out of the way. Carefully dry the surface, and with a camel's-hair pencil apply a free coating of collodion over the wound; let it dry, and repeat it at discretion.

For greater security, shreds of charpie may now be arranged cross-wise over the wound, after the manner of warp and woof; saturate it with collodion, and when dry repeat the process, until the wound is securely cemented over. As a still greater protection, a dossil of lint may then be placed over the part and retained with adhesive straps.

If there be a tendency to undue heat in the part, it may be kept down with cold affusion; should any loosening of the dressing occur, an additional coating of collodion may be applied. The sutures must not be removed until healing by first intention is complete.

Should suppuration occur, so as to occasion distressing dyspnœa, proceed to treat it in all respects as a case of empyema, introducing the trocar at the most dependent point, and taking special care to avoid the admission of air."

Dr. Howard describes particularly three advantages which are gained by this perfect closure of the wound. 1st. Hemorrhage is controlled. At the worst, he says, the amount of blood lost after the operation cannot be more than would suffice to fill up the unoccupied space remaining in the pleural cavity; the elastic clot resulting furnishing a styptic *par excellence* for the wounded vessels of the yielding lung. 2d. Dyspnœa is immediately relieved upon removal of the atmospheric pressure. 3d. Suppuration, if not prevented is greatly diminished by shutting out the constantly renewed currents of atmospheric air, and its character is very favorably modified. "Indeed, if the wound were closed soon enough," says Dr. Howard, "I deem it possible that the slough of the track through the lung, with the limited amount of attendant pus, might be entirely disposed of by absorption and expectoration."

As a proof of the successful results of the sealing plan of treatment, Dr. Howard mentions that some cases upon which he operated were six days in the ambulances before reaching a general hospital, part of the road traveled over being of the worst description: on the fifth day all but one of these so treated were able to walk comfortably.—*London Lancet, March*, 1864.

[Professor Longmore's remarks on this subject will appear in our next number.]

